# Determinants of Quality of Life for Breast Cancer Patients in Shanghai, China

**DOI:** 10.1371/journal.pone.0153714

**Published:** 2016-04-15

**Authors:** Bei Yan, Li-Ming Yang, Li-Peng Hao, Chen Yang, Lei Quan, Li-Hong Wang, Zheng Wu, Xiao-Pan Li, Yu-Tang Gao, Qiao Sun, Jian-Min Yuan

**Affiliations:** 1 Shanghai Pudong New Area Center for Disease Control and Prevention, Shanghai, China; 2 Division of Cancer Control and Population Sciences, University of Pittsburgh Cancer Institute, Pittsburgh, Pennsylvania, United States of America; 3 School of Bioscience and Bioengineering, South China University of Technology, Guangzhou, China; 4 Department of Epidemiology, Shanghai Cancer Institute, Renji Hospital, Shanghai Jiaotong University School of Medicine, Shanghai, China; 5 Department of Epidemiology, Graduate School of Public Health, University of Pittsburgh, Pittsburgh, Pennsylvania, United States of America; Sudbury Regional Hospital, CANADA

## Abstract

**Purpose:**

To evaluate the association of social support status, health insurance and clinical factors with the quality of life of Chinese women with breast cancer.

**Methods:**

Information on demographics, clinical characteristics, and social support status was collected from 1,160 women with newly diagnosed breast cancer in Shanghai, China. The Perceived Social Support Scale was used to assess different sources of social support for breast cancer patients. The quality of life was evaluated using the Functional Assessment of Cancer Therapy-Breast Cancer that consisted of five domains: breast cancer-specific, emotional, functional, physical, and social & family well-being. Multivariate linear regression models were used to evaluate the associations of demographic variables, clinical characteristics, and social support status with the quality of life measures.

**Results:**

Adequate social support from family members, friends and neighbors, and higher scores of Perceived Social Support Scale were associated with significantly improved quality of life of breast cancer patients. Higher household income, medical insurance plans with low copayment, and treatment with traditional Chinese medicine for breast cancer all were associated with higher (better) scores of quality of life measures whereas patients receiving chemotherapy had significantly lower scores of quality of life.

**Conclusion:**

Social support and financial aids may significantly improve the quality of life of breast cancer survivors.

## Introduction

Breast cancer is the most frequently diagnosed malignancy among women. It is estimated that 1.67 million women were diagnosed with breast cancer worldwide in 2012 [1]. During the past 30 years, the incidence rate of breast cancer has been increasing in most countries [1]. In China, the age-standardized incidence rate of breast cancer increased by five times, from 6.4 per 100,000 in 1980 to 31.93 per 100,000 in 2011 [2]. In general patients with breast cancer have a better outcome and longer survival after cancer diagnosis compared to women with other types of cancer [3]. It is estimated that there were more than 697,000 breast cancer survivors in China during 2007–2012 [1]. Therefore, to improve the quality of life of breast cancer survivors in China and elsewhere in the world would have significant social and public health implications.

The quality of life (QOL) is a multidimensional, multifaceted measure that includes the impact of the diagnosis, treatment and progression of the disease on the daily living and rehabilitation of patients with breast cancer [4–5]. More recently, QOL has been viewed as a primary endpoint measure for the quality of the management and care in the oncology medicine. QOL is a subjective assessment of physical, psychological, and social well-being [5–6], and reflects patients’ perceptions of the impact of breast cancer diagnosis and treatment on daily living [7–9]. Numerous studies found that a better QOL measure is associated with longer survival of patients with various types of cancer [10–12]. Thus the identification of determinants for QOL measure may provide insights on the management and care for patients with breast cancer. Several studies reported that age at cancer diagnosis [13–17], level of education [13–15], income [18–20], marital status [15–17,20], the time period since cancer diagnosis [14], and chemotherapy [14,16,19,21] are associated with the QOL measure of breast cancer survivors. In more recent years, social support has been recognized as an important determinant in QOL of cancer survivors independent of types of cancer [19, 22–24]. Most of these studies were conducted in western countries. These findings may not be relevant and directly applicable to cancer survivors in the developing countries such as China that has distinct social and cultural differences from Western societies. Several hospital-based small-scale studies in Chinese women showed that social support improved QOL of Chinese women with breast cancer [25–27].The present study was part of a research program designed to evaluate the effects of comprehensive social support intervention to improve the QOL of women diagnosed with breast cancer in the last 30 months in Shanghai, China. We comprehensively examined the associations of demographic variables, clinical characteristics of breast cancer, and social support status with measures of QOL in the following five domains: breast cancer-specific well-being (BWB), emotional well-being (EWB), functional well-being (FWB), physical well-being (PWB), and social & family well-being (SWB).

## Materials and Methods

The present analysis was based on the baseline data collected for the research program that evaluates the impact of community-based comprehensive social support intervention on the QOL of breast cancer patients in Shanghai. The program included three components: 1) to assess the current social support status that breast cancer patients perceived and received; 2) to develop a comprehensive social support program (i) that provides patients with newly diagnosed breast cancer the knowledge on the treatment options, rehabilitation, breast cancer recurrence and metastasis, pain management, and breast self-examination through the seminars and consultations; and (ii) that provides family members of breast cancer patients the knowledge about patient care and rehabilitation and the encouragement of family members to accompany with the patient participating in treatment, rehabilitation, seminars and consultation; and 3) that recruits breast cancer patients with at least 5 years of survival after cancer diagnosis who can share their experience in management and recovery through the cancer patient healing club. The healing club is a non-governmental, community-based organization that enrolls cancer patients voluntarily. The mission of the club is to organize social activities and seminars that disseminate medical knowledge about cancer diagnosis and treatment, rehabilitation, and a healthy living after cancer diagnosis. The present analysis was the first part of the research program.

### Participants and Procedures

The information on patients who were recently diagnosed with breast cancer was initially obtained from the Shanghai Cancer Registry, a population-based cancer registry that records all newly diagnosed cancer cases and deaths in the Metropolitan Shanghai, China. The Shanghai cancer registry data have been published in consecutive volumes of the Cancer Incidence in Five Continents by the International Agency for Research on Cancer since 1975 [28]. For the present study, the eligibility criteria include women: 1) who were 20 years of age or older at the initial diagnosis with breast cancer during 1^st^ January 2011 through 31^st^ July 2013; 2) who were residents of the Pudong New District Area of the Metropolitan Shanghai; 3) who underwent mastectomy or lumpectomy for breast cancer; and 4) who were able to complete the survey questionnaire. Women with metastatic breast cancer or Karnofsky Scores (KPS) below 70 were ineligible for the present study. This study was approved by the Institutional Review Board of the Shanghai Pudong New Area Center for Disease Control and Prevention. Written informed consent was obtained from all participants before they were enrolled in the study.

The recruitment of pre-identified eligible breast cancer patients into the study was conducted by the primary care physicians at the local community hospitals, where cancer patients were living nearby and seeking primary medical care. All participating physicians were trained for in-person interview using a structured questionnaire. All study subjects was interviewed by the primary care physicians, who briefly described the goals and procedures of the study and obtained written consent from patients before the interview began. The subject recruitment and all interviews were conducted from 1^st^ May through 30^th^ November of 2013. Of the 1,826 eligible patients, 1,160 were enrolled into the present study (63.7% participation rate). Age and the clinical stage of breast cancer at diagnosis were comparable between patients enrolled and those who did not.

### Data Collection

During the interview, we collected information on demographic characteristics (education, occupation, health insurance, marital status, and household income), treatment method for breast cancer (type of surgery, chemotherapy and traditional Chinese medicine), and social support for patients (family harmony status, interaction with friends or neighbors, participating in the healing club, engaging in peer-patient activities and communications). In China, there are three main health insurance programs run by the government. The Urban Employee Basic Medical Insurance (UEBMI) was implemented beginning in 1998. The plan is a part of the work compensation through the premium contribution by both the employer and the employee. The Urban Resident Basic Medical Insurance (URBMI) is a medical insurance plan for minors, students, disabled, elderly and unemployed residents in the city of Shanghai. The plan was implemented beginning in 2007. The New Rural Cooperative Medical Scheme (NRCMS) is for farmers. The plan is individually paid and supported by the local community and subsided by the government. The information on the characteristics of breast cancer was extracted from the records in the Shanghai Cancer Registry, including date and means of diagnosis, subsite, and clinical stage of the cancer using the TNM system. The clinical stage information was an optional rather than required item by the standard operating procedure of cancer registries, thus missing information on the stage of cancer is allowed. There were 16.8% enrolled patients with missing data on clinical stage of breast cancer at diagnosis.

Social support status of patients was assessed by the revised Perceived Social Support Scale (PSSS) that assessed the extent to which patients with breast cancer received support from different sources [29]. The original scale included 12 items that evaluated perceived support from family, friends, and special others. During the validation of this method, examples were added to help patients understand the special others such as supervisors, colleagues, and relatives. The ratings ranged from 1 (totally disagree) to 7 (totally agree). The 12 individual ratings were summed, resulting in a maximum total score of 84. There were some problems with social support for the patient if the summed total score was below 50, and more serious problems if the score below 32. The Chinese version of the PSSS scale has been validated as being satisfactory in reliability and validities among Chinese cancer patients [30].

The outcome measure was the health-related QOL of breast cancer patients evaluated by the Functional Assessment of Cancer Therapy-Breast Cancer (FACT-B), an instrument designed to measure multidimensional QOL including PWB (7 items), SWB (7items), EWB (6 items), FWB (7 items), and BWB (9 items) [31]. The study subjects were asked to rate their responses to all questions on a scale of 0 (not at all) to 4 (very much) for the truthfulness of each statement describing the well-being characteristics during the previous 7 days. The total FACT-B score (TQOL) was calculated by summing over (unweighted) 5 individual subclass well-being scores; higher scores represented better QOL. The instrument has been extensively validated in different racial/ethnic groups, including Chinese population [31–33]. The scale reliability estimates for each of the FACT-B domains and overall functional index were all acceptable (Cronbach’s alpha ≥0.70 and item-total correlations <0.30) [31,32].

### Statistical Analysis

Demographic characteristics, clinical factors and social support status were summarized using frequencies and percentages for categorical variables, and arithmetic means and their standard deviations (SDs) for continuous variables. Analysis of covariance (ANCOVA) method was used to compare least-squared means of the total and individual QOL scores across different categories of demographics (age at diagnosis, levels of education, marital status, occupation, household income, and types of health insurance), clinical characteristics of breast cancer (stage, type of treatment, and time elapsed since initial cancer diagnosis), and social support status (family harmony, interaction with friends and neighbors, participation in the healing club, participation in peer-patient activities and communications, and the score of PSSS). The parsimonious statistical models were developed using a forward/backward stepwise approach to assess independent effect of patient’s characteristics, clinical factors and social support status on the scores of total and subclass QOL domains. The initial criteria were set at the *P*<0.05 for the entry into and P ≥ 0.10 for removal of a given variable from the model. All categorical variables (education, occupation, marital status, health insurance) were each treated as a set of indicator variables. The final model chosen was based on the largest R-square and the lowest global *P* value, and included variables with significant *P* value only. The original data are available upon request.

Data analyses were conducted using the SPSS 16.0 software package (SPSS Inc. Chicago, USA). All *P* values were 2-sided. The *P* values less than 0.05 were considered statistically significant.

## Results

The present study included 1,160 breast cancer patients. The average age at diagnosis was 57.7 (SD11.5) years (Table 1). A majority of breast cancer patients were married (90.5%), employed (78.4%), and attained an education level higher than the middle school (79.0%). There were 1,122 patients with available information on PSSS. Among them, 89.1% had a PSSS score higher than 50.0. Among 965 patients with available stage information, 375 (38.9%) were diagnosed with stage 0 or stage I cancer whereas only 17 (1.8%) with stage IV cancer. Approximately 89% of the patients underwent mastectomy and the remaining 11% lumpectomy. More than half the study population (64.5%) received the treatment of traditional Chinese medicine for breast cancer whereas only 16.5% patients received chemotherapy.

**Table 1:** Characteristics of breast cancer patients (N = 1160) in Shanghai, China, 2011–2013.

Characteristic*	N†	%	Mean ±SD
**Age (year)**			57.72±11.46
≤44	135	11.6	
45–54	304	26.2	
55–64	437	37.7	
≥65	284	24.5	
**Education**			
Primary school or less	243	21	
Middle school and high school	738	63.7	
College and above	177	15.3	
**Marital status**			
Married	1044	90.5	
Unmarried	11	1	
Widowed	73	6.3	
Divorced	26	2.3	
**Occupation**			
Government, authority for the enterprises and public-sector organizations	289	24.9	
Private enterprise and freelance	621	53.5	
Farmers and unemployed	250	21.6	
**Monthly household income (RMB)**			
<1000	56	4.9	
1001–3000	428	37.5	
3001–5000	370	32.4	
>5000	287	25.2	
**Type of health insurance**			
URBMI and NRCMS	181	15.7	
UEBMI	946	81.8	
Undefined	29	2.5	
**Family harmony status**			
Not so good	172	14.9	
Good	985	85.1	
**Interaction with friends or neighbors**			
Never	43	3.7	
Sometimes	447	38.5	
Frequently	670	57.8	
**Participation in healing club**			
No	1051	90.7	
Yes	108	9.3	
**Participation in peer-patient activities and communication**			
No	893	77.1	
Yes	265	22.9	
**Score of PSSS**			65.1±10.4
<50	88	7.8	
50–69	598	53.3	
≥70	436	38.9	
Family support score of PSSS			23.1±3.4
Friend support score of PSSS			20.7±4.2
Other support score of PSSS			23.1±4.0
**Stage of cancer**			
0-I	375	32.3	
II	450	38.8	
III	123	10.6	
IV	17	1.5	
Undefined	195	16.8	
**Surgery**			
Lumpectomy	125	10.8	
Mastectomy	1030	89.2	
**Chemotherapy status**			
No	966	83.5	
Yes	191	16.5	
** Tradition Chinese medicine**			
No	412	35.5	
Yes	748	64.5	
**Time since diagnosis(month)**			15.0±6.7
<11.9	458	39.5	
12.0–23.9	563	48.5	
≥24.0	139	12	
**Breast self-examination**			
No	447	38.5	
Yes	713	61.5	

* NRCMS: new rural cooperative medical scheme. UEBMI: urban employee basic medical insurance. URBMI: urban resident basic medical insurance. PSSS: perceived social support scale.

† The sum of the numbers for some characteristic variables is less than the total (1160) due to missing values.

The associations between QOL measures and demographic characteristics are shown in Table 2. Age at interview (*P* = 0.029), level of education (*P* = 0.001), occupation (*P* <0.001), household income (*P* <0.001), and type of health insurance (*P* <0.001) were significantly associated with TQOL in univariate analysis. Married women had significantly higher QOL score of SWB, but lower score of BWB.

**Table 2:** Quality of life measures by sociodemographic characteristics in breast cancer patients in Shanghai, China, 2011–2013.

Socio-demographic characteristics	PWB*	SWB	EWB	FWB	BWB	TQOL
	Mean ±SD	Mean ±SD	Mean ±SD	Mean ±SD	Mean ±SD	Mean ±SD
**Age(years)**						
< = 44	22.49±4.96	17.94±5.64	17.60±4.31	14.87±6.31	24.53±4.77	97.40±18.07
45–54	22.53±4.70	17.67±5.86	17.67±4.08	14.37±6.24	24.67±4.25	96.91±18.17
55–64	21.92±4.92	16.51±5.94	17.32±4.37	13.16±6.21	24.91±4.12	93.82±19.22
> = 65	22.13±4.58	15.67±5.54	17.49±4.02	12.70±5.93	25.60±3.98	93.58±17.60
**P value**	0.315	**<0.001**	0.709	**<0.001**	**0.024**	**0.029**
**Education**						
Primary school or less	22.55±4.46	15.16±5.96	16.69±4.17	12.31±5.99	24.85±4.41	91.06±17.91
Middle school and high school	22.29±4.75	17.03±5.68	17.68±4.00	13.66±6.06	25.05±4.08	95.72±17.99
College and above	22.65±5.29	17.26±6.12	17.78±4.88	14.85±6.74	24.80±4.46	97.33±20.44
**P value**	**0.043**	**0.003**	**0.004**	**<0.001**	0.694	**0.001**
**Marital status**						
Married	22.31±4.63	17.02±5.80	17.55±4.07	13.59±6.20	24.83±4.16	95.30±18.28
Single	20.82±7.53	14.55±4.74	17.91±4.89	11.82±7.85	26.64±5.07	91.73±22.27
Widowed	21.18±5.80	15.49±5.65	16.42±5.61	12.97±5.81	26.14±4.36	92.21±20.30
Divorced	20.96±6.43	11.12±5.57	16.50±3.97	13.92±6.69	25.88±4.74	88.38±18.65
**P value**	0.091	**<0.001**	0.092	0.657	**0.022**	0.133
**Occupation**						
Government, authority for the enterprises and public-sector organizations	22.74±4.85	18.03±5.82	18.01±4.12	14.42±5.94	24.83±4.22	98.03±18.23
Private enterprise and freelance	22.27±4.70	16.05±5.70	17.74±3.91	13.49±6.24	25.36±4.07	94.90±18.30
Farmers and unemployed	21.38±4.84	17.16±6.96	16.24±4.72	12.76±6.30	24.18±4.43	91.71±18.70
**P value**	**0.004**	**<0.001**	**<0.001**	**0.007**	**0.001**	**<0.001**
**Monthly household income(RMB)**						
<1000	19.79±5.23	15.36±6.44	15.41±4.90	10.37±5.94	23.73±4.89	84.66±19.14
1001–3000	21.53±4.88	16.58±6.01	16.81±4.46	12.95±6.34	24.50±4.44	92.37±19.20
3001–5000	22.16±4.82	16.34±5.54	17.62±3.36	13.92±6.06	24.74±4.14	94.78±17.95
>5000	23.71±3.91	17.80±5.79	18.71±3.59	14.49±5.99	26.13±3.52	100.841±16.37
**P value**	**<0.001**	**0.002**	**<0.001**	**<0.001**	**<0.001**	**<0.001**
**Type of health insurance**						
URBMI and NRCMS	20.65±4.45	16.15±6.09	15.61±4.48	11.30±6.08	23.87±4.54	87.58±17.34
UEBMI	22.53±4.76	16.91±5.81	17.83±4.06	14.01±6.15	25.17±4.14	96.45±18.38
Undefined	21.66±5.16	16.59±4.80	18.21±3.52	13.03±5.86	25.31±3.04	94.79±17.57
**P value**	**<0.001**	**0.272**	**<0.001**	**<0.001**	**0.001**	**<0.001**
**Total**	**22.19±4.79**	**16.78±5.84**	**16.48±4.20**	**13.56±6.21**	**24.97±4.21**	**94.99±18.48**

* BWB: breast cancer-specific well-being. EWB: emotional well-being. FWB: functional well-being. PWB: physical well-being. SWB: social & family well-being. TQOL: total quality of life score. NRCMS: new rural cooperative medical scheme. UEBMI: urban employee basic medical insurance. URBMI: urban resident basic medical insurance.

The associations between clinical factors and QOL measures are shown in Table 3. Patients receiving traditional Chinese medicine as a treatment for breast cancer reported higher scores of all QOL domains (all *Ps*<0.01) whereas those receiving chemotherapy reported lower scores of PWB, EWB, BWB and TQOL (all *Ps*<0.001).

**Table 3:** Quality of life measures by clinical factors in breast cancer patients in Shanghai, China, 2011–2013.

Clinical factor	PWB*	SWB	EWB	FWB	BWB	TQOL
	Mean ±SD	Mean ±SD	Mean ±SD	Mean ±SD	Mean ±SD	Mean ±SD
**Stage at diagnosis**						
0-I	21.89±4.98	16.55±5.81	17.54±4.26	13.45±6.29	24.90±4.04	94.33±18.89
II	22.29±4.68	16.66±5.93	17.22±4.37	13.36±6.30	24.98±4.46	94.52±19.02
III	22.37±4.85	17.63±5.90	17.53±4.34	13.85±5.69	25.146±4.22	96.53±17.80
IV	22.06±5.47	16.71±5.67	17.35±2.12	13.12±5.36	23.88±3.62	96.12±14.10
Undefined	22.46±4.56	16.96±5.66	17.96±3.86	14.09±6.18	25.09±3.99	96.56±17.11
**P value**	0.645	0.463	0.365	0.669	0.807	0.523
**Surgical treatment**						
Lumpectomy	22.33±4.79	16.70±5.09	17.33±4.53	13.82±6.62	24.76±4.52	94.94±18.46
Mastectomy	22.18±4.78	16.77±5.93	17.50±4.16	13.51±6.15	25.00±4.17	94.97±18.49
**P value**	0.75	0.898	0.659	0.606	0.555	0.985
**Chemotherapy status**						
No	22.63±4.54	16.81±5.85	17.68±4.10	13.71±6.25	25.34±4.05	96.16±18.21
Yes	19.98±5.42	16.68±5.77	16.50±4.58	12.87±5.92	23.07±4.49	89.11±18.81
**P value**	**<0.001**	0.784	**<0.001**	0.088	**<0.001**	**<0.001**
**Tradition Chinese medicine**						
No	21.37±5.15	16.08±5.82	16.64±4.37	12.88±6.37	24.30±4.35	91.28±18.95
Yes	22.65±4.52	17.16±5.82	17.95±4.03	13.94±6.08	25.34±4.09	97.04±17.90
**P value**	**<0.001**	**0.003**	**<0.001**	**0.006**	**<0.001**	**<0.001**
**Time since diagnosis(month)**						
<11.9.	21.41±4.91	16.77±5.65	17.11±4.13	12.86±5.99	24.59±4.31	92.75±17.86
12.0–23.9	22.70±4.78	16.72±5.97	17.77±4.28	13.98±6.22	25.32±4.08	96.48±18.70
> = 24.0	22.73±4.02	17.04±5.94	17.58±4.04	14.18±6.62	24.81±4.26	96.33±18.95
**P value**	**<0.001**	0.841	**<0.045**	**0.008**	**0.019**	**0.004**
**Breast self-examination**						
No	21.92±4.88	16.31±5.81	17.19±4.48	12.30±6.16	24.94±4.13	92.66±19.39
Yes	22.37±4.72	17.07±5.84	17.67±4.00	14.35±6.10	24.99±4.26	96.45±17.74
**P value**	0.124	**0.031**	0.057	**<0.001**	0.834	**0.001**

* BWE: breast cancer-specific well-being. EWB: emotional well-being. FWB: functional well-being. PWB: physical well-being. SWB: social & family well-being. TQOL: total quality of life score.

The associations between social support status and the measures of QOL are shown in Table 4. Breast cancer patients with good family harmony and frequent interaction with friends and neighbors reported significantly higher QOL scores of all domains of FACT-B. The participation in the healing club was associated with higher QOL scores of all but PWB. The level of PSSS was positively and significantly associated with higher QOL score of all domains except for BWB.

**Table 4:** Quality of life measures by social support status in breast cancer patients in Shanghai, China, 2011–2013.

Social support status	PWB*	SWB	EWB	FWB	BWB	TQOL
	Mean ±SD	Mean ±SD	Mean ±SD	Mean ±SD	Mean ±SD	Mean ±SD
**Family harmony status**						
Not so good	20.01±5.31	12.65±5.10	14.94±4.44	10.76±5.71	23.95±4.64	82.30±17.60
Better	22.57±4.59	17.50±5.67	17.92±4.00	14.06±6.16	25.15±4.11	97.21±17.75
**P value**	**<0.001**	**<0.001**	**<0.001**	**<0.001**	**0.002**	**<0.001**
**Interaction with friends or neighbors**						
Never	20.05±6.00	11.51±5.88	14.42±6.07	9.51±6.17	24.35±4.15	79.84±21.03
Sometimes	21.35±4.88	14.40±5.33	16.43±4.27	11.55±5.69	24.52±4.16	88.25±17.88
Frequently	22.90±4.51	18.70±5.36	18.39±3.73	15.16±6.03	25.31±4.22	100.47±16.60
**P value**	**<0.001**	**<0.001**	**<0.001**	**<0.001**	**0.005**	**<0.001**
**Participation in healing club**						
No	22.16±4.81	16.53±5.82	17.36±4.17	13.40±6.23	25.06±4.18	94.51±18.50
Yes	22.44±4.61	19.15±5.46	18.63±4.32	15.04±5.71	24.12±4.45	99.38±17.64
**P value**	**0.563**	**<0.001**	**0.003**	**0.009**	**0.028**	**0.009**
**Participation in peer-patient activities and communication**						
No	22.13±4.91	16.24±5.86	17.22±4.26	13.11±6.27	25.03±4.18	93.73±18.91
Yes	22.39±4.37	18.58±5.39	18.36±3.88	15.11±5.68	24.76±4.31	99.20±16.21
**P value**	**0.431**	**<0.001**	**<0.001**	**<0.001**	**0.357**	**<0.001**
**Score of PSSS**						
<50	21.22±5.54	10.65±5.38	15.21±5.40	9.98±6.15	25.24±4.54	82.20±20.36
50–69	21.70±4.83	15.23±5.05	17.01±4.14	12.26±5.70	24.85±4.06	91.06±17.40
> = 70	22.95±4.51	20.43±4.71	18.65±3.30	16.32±5.88	25.03±4.41	103.39±16.48
**P value**	**<0.001**	**<0.001**	**<0.001**	**<0.001**	**0.642**	**<0.001**

* BWB: breast cancer-specific well-being. EWB: emotional well-being. FWB: functional well-being. PWB: physical well-being. SWB: social & family well-being. TQOL: total quality of life score. PSSS: perceived social support scale.

Table 5 shows the results of multivariable regression analysis for overall and each domain QOL after adjustment for each other. Higher household income was associated with higher QOL scores of all domains. Compared with married women, single or divorced women had lower SWB, but higher BWB. Patients with the UEBMI plans had higher scores of several QOL. Women receiving chemotherapy for breast cancer had lower QOL whereas those receiving traditional Chinese medicine reported higher QOL scores. Frequent interaction with friends and neighbors was associated with higher scores in all domains of QOL. Familial harmony was associated with better measures of PWB, SWB and TQOL. TQOL score was increased by 5.54 per 10 points of PSSS.

**Table 5:** Multivariate regression coefficients of socio-demographic characteristics, clinical factors, and social support status on quality of life measures in breast cancer patients in Shanghai, China, 2011–2013.

Characteristics	PWB*		SWB		EWB		FWB		BWB		TQOL	
	β	*p*	β	*p*	β	*p*	β	*p*	β	*p*	β	*p*
**Age (per 10 years)**	-0.10	0.408	-0.54	**<0.001**	0.03	0.770	-0.44	**0.002**	0.38	**0.001**	-0.62	0.132
**Time since diagnosis(month)**	0.07	**0.001**	0.04	**0.032**	0.05	**0.004**	0.10	**<0.001**	0.02	0.337	0.27	**<0.001**
**Marital status**												
Married	/†	/		Ref		/	/	/	/	Ref	/	/
Single	/	/	-1.36	0.316	/	/	/	/	2.62	**0.035**	/	/
Widowed	/	/	-0.23	0.682	/	/	/	/	1.05	**0.041**	/	/
Divorced	/	/	-4.19	**<0.001**	/	/	/	/	1.76	**0.031**	/	/
**Occupation**												
Government, authority for the enterprises and public-sector organizations	/	/	/	/	1.09	**0.007**	/	/	/	/	/	/
Private enterprise and freelance	/	/	/	/	0.91	**0.013**	/	/	/	/	/	/
Farmers and unemployed	/	/	/	/	Ref		/	/	/	/	/	/
**Monthly household income (category)‡**	0.59	**<0.001**	0.29	**0.017**	0.30	**0.005**	0.77	**<0.001**	0.55	**<0.001**	2.15	**<0.001**
**Type of health insurance**												
URBMI and NRCMS	/	/	Ref		Ref		Ref		/	/	Ref	
UEBMI	/	/	0.90	**0.018**	1.48	**<0.001**	2.59	**<0.001**	/	/	7.67	**<0.001**
Undefined	/	/	0.60	0.510	2.30	**0.004**	1.45	0.194	/	/	6.28	0.056
**Chemotherapy status (yes)**	-0.97	**<0.001**	/	/	/	/	/	/	-0.53	**<0.001**	-2.21	**0.006**
**Tradition Chinese medicine (yes)**	0.95	**0.001**	/	/	0.76	**0.002**	/	/	1.09	**<0.001**	2.81	**0.004**
**Breast self-examination (yes)**	/	/	/	/	/	/	0.83	**0.016**	/	/	/	/
**Family harmony status (yes)**	0.84	**<0.001**	0.552	**0.040**	0.87	**<0.001**	/	/	/	/	3.21	**0.001**
**Interaction with friends or neighbors (yes)**	1.11	**<0.001**	2.29	**<0.001**	1.47	**<0.001**	2.27	**<0.001**	0.62	**0.002**	7.90	**<0.001**
**Score of PSSS (per 10 units)**	0.37	**0.008**	2.69	**<0.001**	0.82	**<0.001**	1.71	**<0.001**	/	/	5.54	**<0.001**

* BWB: breast cancer-specific well-being. EWB: emotional well-being. FWB: functional well-being. PWB: physical well-being. SWB: social & family well-being. TQOL: total quality of life score. NRCMS: New rural cooperative medical scheme. UEBMI: urban employee basic medical insurance. URBMI: urban resident basic medical insurance. PSSS: perceived social support scale.

† the symbol “/” means the factor was not included in the final regression model for this domain based on the stepwise approach with the entry p ≤ 0.05 and removal p ≥ 0.10, except for age and time since diagnosis, which were forced into all regression models.

‡ See the categorical values in Table 1.

## Discussion

The present study demonstrates that income, social support and treatment for breast cancer have significant impact on quality of life of Chinese women with breast cancer. These findings provide a scientific basis to develop a comprehensive program that incorporates these factors, especially social support, to improve the QOL of breast cancer survivors in China as well as elsewhere in the world. In addition, the consistent results of the present study in a Chinese population with those in Western population support a universal effect of age, income, marital status, social support, and chemotherapy on the long-term quality of life of breast cancer patients [19–21].

The present study shows that high household income is associated with a better QOL of patients with breast cancer in each and every domain measured. Our findings are consistent with those of previous studies [18–20,34,35]. Higher socioeconomic status has been linked to many aspects of better care of patients such as the prompt treatment, access to comprehensive rehabilitation, taking leave of absence from work and having less worry about financial constraint [36,37]. The type of health insurance is directly related to the financial stress of a patient after cancer diagnosis in the present study population. The coverage for medical expenses by UEBMI is higher than by URBMI and NRCMS. Compared with URBMI and NRCMS, patients in UEBMI had better measures of SWB, EWB, FWB and TQOL. The difference could be related to the different financial risk and burden of medical care cost due to breast cancer under different medical insurance plans. Some studies showed that NRCMS has higher financial risk and burden of medical care cost than UEBMI [38]. Based the findings of the present study, financial support for patients who are recently diagnosed with breast cancer through governmental subsidies and other supporting mechanisms may significantly improve their quality of life and recovery from their breast cancer.

Similar to previous studies, the present study demonstrates that chemotherapy is associated with lower score of overall QOL measure, possibly related to its toxicity and severe side-effects [16,21]. Interestingly traditional Chinese medicine can significantly improve the quality of life of breast cancer patients in several domains including the physical, emotional, and breast cancer-specific well-being even after adjustment for chemotherapy, socioeconomic and social support status. Several studies found that adjuvant therapy of traditional Chinese medicine was effective in alleviating the cancer-related symptoms and chemotherapy-related side effects including diarrhea and poor appetite [39,40], and may even reduce the risk of death from advanced breast cancer [41]. The findings of the present study, if confirmed by other studies, may be incorporated in a medical care and management program for breast cancer patients, especially those who are receiving chemotherapy.

Social support relies on a multidimensional supportive network with both quantity of social ties and the quality of relationship with other people who provide support for the patients. The same social network would have different effect on different patients because of their difference in personal characteristics. Thus, it is a challenge to comprehensively evaluate individual social support status. The dimensions of social support include perceived availability of support, actual support received, and reciprocity, i.e., the provision and receiving support [42]. In the present study, we used a validated comprehensive measure for social support, the PSSS, to assess the extent to which breast cancer survivors currently perceived support from different sources, which evaluates the social support status dependent on the self-feeling of a given patient. Thus, the PSSS assesses the individual social support status more objectively than the measure based on her social network. Our study demonstrates that the scores of PSSS are significantly and positively associated with the QOL measures of breast cancer patients in a dose-dependent manner. Our findings are consistent with those reported by Candyce and coworkers who used a social network approach that assessed both quantity of social ties and quality of relationship [43]. Good status of family harmony and frequent interactions with friends and neighbors, two specific measures of social support, significantly improve the QOL of breast cancer survivors. Divorced women had a statistically significant 30% lower score of SWB compared with married women with breast cancer. These findings strongly implicate that social support from family members and friends as well as other social connections plays an important role in coping with and recovering from breast cancer. Social support should be a key component of a management and care of breast cancer survivors.

In the present study, after adjustment for other factors, participation in the cancer patient healing club had no additional beneficial effect on QOL of breast cancer patients. Participation in the healing club is highly correlated with the participation in the peer-patient activities and communications (the correlation coefficient = 0.49, *P*<0.001). Given that the latter represents a more comprehensive measure for social support, it has a stronger effect on QOL measures than the healing club. When both variables were simultaneously included in the logistic model, the effect of participation in the healing club on QOL was no longer statistically significant (*P =* 0.345) whereas the impact of the participation in the peer-patient activities and communications on patients’ QOL remained statistically significant (*P* = 0.001).

The present study has several strengths. The study used a novel, comprehensive approach for assessment of social support. The validated standardized scales for QOL assessment can allow for the direct comparison with other studies conducted in Chinese women [14,44]. A larger sample size provided sufficient statistical power to simultaneously evaluate the independent effect of many factors that may have impact on QOL measures. There also are several limitations in the present study. We did not collect information on co-morbidities of patients (such as depression, diabetes, and other chronic conditions), physical excise and the breast reconstruction after mastectomy. All these factors may have impact on the QOL measures [45,46]. Because this study was used a cross-sectional study design, the causal effect of the social support and traditional Chinese medicine on patients’ QOL cannot be inferred. In addition, we did not collect information on the QOL measure of women before their diagnosis of breast cancer, thus were unable to examine the change of QOL before and after the diagnosis of breast cancer. The majority of patients in the study were married, well educated, diagnosed with an early stage breast cancer and well insured. All of these factors would have a positive impact on the prognosis. Thus findings of the present study may not be directly applicable to patients in rural areas of China or other patient populations in Western societies, where there is significant difference in cultural, societal and familial characteristics.

In summary, the present study demonstrates that high household income and adequate social support significantly and independently improve QOL of Chinese women with breast cancer. These findings, if confirmed by other studies, support a comprehensive program with financial and social support components for the management and care of breast cancer survivors. The future studies are warranted to further examine and confirm the beneficial effect of traditional Chinese medicine on the improvement of QOL in patients with breast cancer. If confirmed, the remedy can be introduced to western societies that currently lack or have limited access to such remedy.
